# Determinants of hospital length of stay for people with serious mental illness in England and implications for payment systems: a regression analysis

**DOI:** 10.1186/s12913-015-1107-6

**Published:** 2015-09-30

**Authors:** Rowena Jacobs, Nils Gutacker, Anne Mason, Maria Goddard, Hugh Gravelle, Tony Kendrick, Simon Gilbody

**Affiliations:** 1 Centre for Health Economics, University of York, Heslington, York, UK; 2 Primary Care and Population Sciences, University of Southampton, Southampton, UK; 3 Department of Health Sciences, University of York, Heslington, York, UK

**Keywords:** Schizophrenia, Bipolar disorder, Psychosis, Serious mental illness, Length of stay, Hospitalisation, Mental health funding, Prospective payment, Resource use

## Abstract

**Background:**

Serious mental illness (SMI), which encompasses a set of chronic conditions such as schizophrenia, bipolar disorder and other psychoses, accounts for 3.4 m (7 %) total bed days in the English NHS. The introduction of prospective payment to reimburse hospitals makes an understanding of the key drivers of length of stay (LOS) imperative. Existing evidence, based on mainly small scale and cross-sectional studies, is mixed. Our study is the first to use large-scale national routine data to track English hospitals’ LOS for patients with a main diagnosis of SMI over time to examine the patient and local area factors influencing LOS and quantify the provider level effects to draw out the implications for payment systems.

**Methods:**

We analysed variation in LOS for all SMI admissions to English hospitals from 2006 to 2010 using Hospital Episodes Statistics (HES). We considered patients with a LOS of up to 180 days and estimated Poisson regression models with hospital fixed effects, separately for admissions with one of three main diagnoses: schizophrenia; psychotic and schizoaffective disorder; and bipolar affective disorder. We analysed the independent contribution of potential determinants of LOS including clinical and socioeconomic characteristics of the patient, access to and quality of primary care, and local area characteristics. We examined the degree of unexplained variation in provider LOS.

**Results:**

Most risk factors did not have a differential effect on LOS for different diagnostic sub-groups, however we did find some heterogeneity in the effects. Shorter LOS in the pooled model was associated with co-morbid substance or alcohol misuse (4 days), and personality disorder (8 days). Longer LOS was associated with older age (up to 19 days), black ethnicity (4 days), and formal detention (16 days). Gender was not a significant predictor. Patients who self-discharged had shorter LOS (20 days). No association was found between higher primary care quality and LOS. We found large differences between providers in unexplained variation in LOS.

**Conclusions:**

By identifying key determinants of LOS our results contribute to a better understanding of the implications of case-mix to ensure prospective payment systems reflect accurately the resource use within sub-groups of patients with SMI.

**Electronic supplementary material:**

The online version of this article (doi:10.1186/s12913-015-1107-6) contains supplementary material, which is available to authorized users.

## Background

Serious mental illness (SMI) encompasses a range of chronic and frequently disabling conditions including schizophrenia, bipolar disorder and psychoses. These conditions are associated with substantial morbidity and mortality. The life expectancy of SMI patients is 10 to 15 years shorter than the general population in England [1], and 15 to 20 years shorter in Denmark, Finland and Sweden [2]. A recent global morbidity study attributed 3.5 % of total Years Lost to Disability to schizophrenia and bipolar disorder combined [3]. The two diseases alone are estimated to constitute 1.5 % of the total Disability Adjusted Life Year burden of disease for the UK in 2010 [4] and 1.1 % in 21 regions worldwide [5]. People with SMI are at higher risk of hospitalisations than the general population [6, 7] as physical comorbidity is more common [8, 9]. SMI is associated with increased treatment costs [10] and hospitalisation for this patient group represents a significant proportion of health care resource use. In England, these illnesses account for 3.4 million or 7.2 % of total bed days [11]. This paper examines the key patient and local area determinants of inpatient length of stay (LOS) for patients with a main diagnosis of SMI and examines the variation in LOS between hospital providers in England.

The delivery of mental health services and the incentives that service providers face have changed radically in the last few decades. Most western health care systems have deinstitutionalised care for patients with mental health problems and shifted treatment from secondary care settings into the community [12]. This has led to significant reductions in average LOS and also in overall numbers of psychiatric beds. More recently, policy shifts have focused on changes in funding arrangements for mental health care as a response to pressure to contain costs. Whereas most health care systems reimburse the full costs for providers of inpatient care, several are considering the use of activity-based prospective payment systems, similar to those already in use in the acute physical care setting, in order to reduce costs [13]. Canada (Ontario), Australia and New Zealand have developed case-mix classification systems for mental health services which have included information on diagnosis. In Australia and New Zealand provider factors were shown to significantly drive cost variations making the classification systems unsuitable for provider payment [13].

In England, the National Health Service (NHS) is moving away from traditional block contracts towards a more transparent prospective funding for providers called the National Tariff Payment System (NTPS) (formerly known as Payment by Results (PbR) [14]). Under the NTPS, patients are classified into one of 21 care clusters based on need and severity, rather than diagnostic coding. These clusters are in turn grouped into three super-classes corresponding to non-psychotic, psychosis and organic mental illness. The intention is that each cluster will have a fixed national price based on the national weighted average cost of admitted and non-admitted care. Each cluster has a specific review period attached to it with payments made to cover all care during the cluster review period. Whilst the current implementation of NTPS focuses on the development of locally negotiated cluster prices, the move towards a national fixed price payment system would provide a strong incentive to control costs and should therefore encourage providers to reduce LOS. Evidence from the US has reported reductions in LOS following the introduction of a prospective payment system in psychiatric care, as well as reductions in LOS due to anticipatory effects prior to payments starting [15, 16]. LOS for inpatient care is a major driver of resource use and is highly correlated with hospital costs, especially when care is labour-intensive as is the case in mental health [17]. Reductions in LOS may reduce the very high psychiatric bed occupancy rates observed in the English NHS and the associated difficulties in accessing acute psychiatric beds for severely ill patients in crisis [18], although driving down reductions in LOS too far can impact on quality and outcomes and may increase readmission rates [19–21].

Differences in LOS across providers can reflect differences in patient needs, but can also be indicative of differences in treatment philosophies and practice patterns [22] and in efficiency of care provision. A better understanding of the factors which determine LOS is imperative for the design of payment systems, e.g. by identifying high cost casemix profiles. Estimates of how LOS varies between providers after allowing for differences in case-mix can also provide measures of the extent to which LOS may be amenable to potential reductions by high cost providers in response to the introduction of a prospective payment system. Given the high proportion of bed days and the high cost associated with the care of people with psychotic disorders, as well as the fact that psychosis is one of the three super-classes in the NTPS, this study focuses on the determinants of LOS for people with SMI.

There is conflicting evidence about the key determinants of hospital LOS for people with SMI. This may in part reflect the methodological weaknesses in many previous studies. Many studies are cross-sectional with small samples split into case–control groups by mean or median LOS in order to examine the difference between long and short-stays, typically using logistic regression. Comparing sub-populations in this way leads to inconsistent findings as LOS is typically skewed and sub-populations may be small [12]. Single site studies are not generalisable to other settings with a different patient case-mix [23]. Finally, SMI covers a range of clinical sub-groups with different treatment requirements. Studies to date have typically pooled clinical sub-groups to increase their sample size, making the untested assumption that risk factors will have the same effect on all sub-groups.

This study has two aims. First, we aim to assess the independent effects of patient characteristics (case-mix) and local area characteristics on LOS and study whether there is heterogeneity in those effects across patient sub-groups with SMI. We improve on previous work by using large scale administrative datasets to investigate factors associated with LOS. Second, we aim to assess the degree of unexplained variation in provider LOS i.e. the variation which remains after controlling for the patient and local area characteristics in our model. The residual unexplained variation in LOS may be interpreted as the element most amenable to influence by policymakers and providers. Thus it may help to define the limits on the extent to which a prospective payment system for providers may be successful in reducing LOS and costs.

### Determinants of length of stay for patients with serious mental illness

We searched the literature for key determinants of LOS for patients with SMI to identify a relevant set of explanatory variables for subsequent analysis. We searched several bibliographic databases (e.g. PubMed, EMBASE, PsycINFO) to identify relevant literature published between 1946 and 2014. Our search strategy (see Additional file 1: Appendix 1) included terms for schizophrenia, psychotic disorders, bipolar disorder; for trials, cohort studies or systematic reviews; and length of stay. Titles were screened and abstracts were checked for relevance from 132 articles. We found 15 studies with LOS as the primary or secondary outcome for patients with SMI specifically. We also identified 5 studies from alternative sources such as suggestions from experts.

Most studies consider 3 groups of predictor variables: (a) socio-demographic characteristics of patients (e.g. age, gender, living arrangements, degree of social support, ethnicity, insurance status); (b) clinical characteristics (e.g. psychiatric diagnosis, severity, legal status/compulsory admission, psychiatric or physical co-morbidities, measure of functioning, previous admissions, medication); and (c) characteristics of hospitals or the health care system (e.g. type of hospital, measures of quality of care).

While some studies covered a wide array of determinants, many of these were found not to be significant and the results for some factors differed across studies. Socio-demographic characteristics which were associated with increased LOS for patients with SMI include being single / not married [24–26], having accommodation or housing problems [12, 26–28], having no educational qualification [12, 29], being on a national health insurance plan [30, 31], and being in receipt of welfare [29], whilst higher deprivation was associated with shorter LOS in another study [32]. There is limited evidence of an effect for ethnicity [25]. Being a foreigner was associated with increased LOS in one study [29] while being a migrant was associated with reduced LOS in another [12]. Having family ties or social support was also associated with reduced LOS [33, 34]. Older age was associated with increased LOS in some studies [25, 30, 32, 33, 35], and reduced LOS in others [29, 31, 36], while male gender was associated with increased LOS in some studies [24, 30, 31], and reduced LOS in others [25, 26, 32, 37].

Clinical characteristics which were associated with increased LOS for patients with SMI include: a primary diagnosis of schizophrenia or psychosis [25–27, 29, 31, 32, 35, 36, 38, 39] or a mood disorder [35] although some studies found diagnosis to be a poor predictor of LOS [39, 40]. Other characteristics associated with increased LOS were higher severity as measured by e.g. the Brief Psychiatric Rating Scale (BPRS) [24, 41, 42] or the Global Assessment of Functioning (GAF) [37] or other severity indicators [28, 39]. Co-morbidities were associated with increased LOS in some studies [24, 29], while having no secondary diagnoses increased LOS in other studies [30]. A diagnosis of co-morbid substance abuse was associated with a reduced LOS [35, 37, 39] as was personality disorder [37]. Prior hospitalisation was associated with increased LOS in some studies [32, 35, 38] but with lower LOS in other studies [29]. Previous violence / forensic history was positively associated with LOS [28, 33] as was use of seclusion or restraint [12, 37]. Legal status/compulsory admission as a risk factor was positively associated with LOS in some studies [23, 38], but negatively in others [25, 26]. Being on an open rather than a locked ward was associated with reduced LOS [29] as was having an emergency admission or weekend admission [32] and being discharged against medical advice [26]. Receiving psychopharmacological medication, such as neuroleptics, antidepressants and lithium was associated with reduced LOS in one study [29] and increased LOS in another [27]. Being admitted from another institution was positively associated with LOS in one study [34] and negatively in another [12].

Finally, characteristics of hospitals and the healthcare system which were positively associated with LOS include the patient being treated at a psychiatric hospital, rather than another type of hospital [30, 31], a higher number of beds [25, 30, 31], a higher proportion of male patients [31], and a higher proportion of elderly patients [31]. The number of health care professionals employed was associated with reduced LOS [30, 31] as was a shorter distance from patient’s place of residence to hospital [24]. There was also evidence of marked regional variation in LOS [12, 38].

## Methods

### Study population

Our study population was all patients aged 18 or over and admitted with a primary diagnosis of SMI to a mental health hospital in England during the study period April 2006 to October 2010. All patients were followed until March 2011. SMI patients were identified using ICD-10 diagnostic codes in the primary diagnosis field of their admission record. Many studies focus on a wide range of mental health conditions and thus tend to group the primary diagnoses according to type of disorder by ICD-10 code (e.g. F2, F3) which also reflects severity to some degree [12, 43]. We focussed on individual conditions within SMI to more accurately assess the impact on resource use for each condition. In addition to considering the effects of patient and local area characteristics on LOS for all SMI patients in a pooled model (1), we also examined patients with three types of SMI: (2) schizophrenia (F20); (3) schizoaffective disorders, and schizotypal and delusional disorders (F21- F29); and (4) bipolar and mood affective disorders (F30-F31) (see Table 1).

**Table 1:** Descriptive statistics for admissions contributing to the regression analyses

Variable	Pooled (*N* = 89,510) (1)		Schizophrenia (*N* = 38,216) (2)		Psychotic and schizoaffective disorder (*N* = 21,415) (3)		Manic and bipolar disorder (*N* = 29,879) (4)	
Main diagnosis (n, %)								
Schizophrenia (F20)	38,216	(42.7)	38,216	(100.0)				
Schizotypal disorder (F21)	229	(0.3)			229	(1.1)		
Persistent delusional disorder (F22)	3,605	(4.0)			3,605	(16.8)		
Acute and transient psychotic disorder (F23)	6,446	(7.2)			6,446	(30.1)		
Induced delusional disorder (F24)	66	(0.1)			66	(0.3)		
Schizoaffective disorders (F25)	8,200	(9.2)			8,200	(38.3)		
Other nonorganic psychotic disorders (F28)	268	(0.3)			268	(1.3)		
Unspecified nonorganic psychosis (F29)	2,601	(2.9)			2,601	(12.1)		
Manic episode (F30)	2,777	(3.1)					2,777	(9.3)
Bipolar affective disorder (F31)	27,102	(30.3)					27,102	(90.7)
Age (n, %)								
Age up to 25	8,224	(9.2)	3,893	(10.2)	2,795	(13.1)	1,536	(5.1)
Age 25-34	17,951	(20.1)	9,213	(24.1)	4,623	(21.6)	4,115	(13.8)
Age 35-44	22,116	(24.7)	10,308	(27.0)	5,094	(23.8)	6,714	(22.5)
Age 45-54	17,997	(20.1)	7,298	(19.1)	3,824	(17.9)	6,875	(23.0)
Age 55-64	11,652	(13.0)	4,194	(11.0)	2,281	(10.7)	5,177	(17.3)
Age 65-74	7,110	(7.9)	2,203	(5.8)	1,402	(6.5)	3,505	(11.7)
Age 75 and over	4,460	(5.0)	1,107	(2.9)	1,396	(6.5)	1,957	(6.5)
Gender (n, %)								
Female	42,589	(47.6)	13,217	(34.6)	11,292	(52.7)	18,080	(60.5)
Male	46,921	(52.4)	24,999	(65.4)	10,123	(47.3)	11,799	(39.5)
Detention status (n, %)								
Not detained	72,273	(80.7)	30,554	(80.0)	17,039	(79.6)	24,680	(82.6)
Detained	17,237	(19.3)	7,662	(20.0)	4,376	(20.4)	5,199	(17.4)
Ethnicity (n, %)								
White	67,980	(75.9)	27,330	(71.5)	15,841	(74.0)	24,809	(83.0)
Mixed	1,822	(2.0)	948	(2.5)	443	(2.1)	431	(1.4)
Asian	6,728	(7.5)	3,290	(8.6)	1,684	(7.9)	1,754	(5.9)
Black	8,898	(9.9)	5,051	(13.2)	2,172	(10.1)	1,675	(5.6)
Unknown or missing	4,082	(4.6)	1,597	(4.2)	1,275	(6.0)	1,210	(4.0)
Patient has a carer (n, %)								
No	83,426	(93.2)	35,647	(93.3)	19,958	(93.2)	27,821	(93.1)
Yes	6,084	(6.8)	2,569	(6.7)	1,457	(6.8)	2,058	(6.9)
Patient was previously treated for mental health issues (n, %)								
No	48,126	(53.8)	19,377	(50.7)	12,803	(59.8)	15,946	(53.4)
Yes	41,384	(46.2)	18,839	(49.3)	8,612	(40.2)	13,933	(46.6)
Alcohol and substance misuse (n, %)								
No	84,786	(94.7)	35,797	(93.7)	20,304	(94.8)	28,685	(96.0)
Yes	4,724	(5.3)	2,419	(6.3)	1,111	(5.2)	1,194	(4.0)
Co-morbid personality disorder (n, %)								
No	88,329	(98.7)	37,800	(98.9)	21,077	(98.4)	29,452	(98.6)
Yes	1,181	(1.3)	416	(1.1)	338	(1.6)	427	(1.4)
Number of comorbidities (mean, sd)	0.43	(1.0)	0.39	(1.0)	0.47	(1.1)	0.45	(1.1)
Discharge type (n, %)								
Discharged by consultant	87,063	(97.3)	37,148	(97.2)	20,790	(97.1)	29,125	(97.5)
Self-discharged	2,017	(2.3)	902	(2.4)	525	(2.5)	590	(2.0)
Died in hospital	430	(0.5)	166	(0.4)	100	(0.5)	164	(0.5)
Resident in urban area (n, %)								
No	8,959	(10.0)	2,782	(7.3)	2,251	(10.5)	3,926	(13.1)
Yes	80,551	(90.0)	35,434	(92.7)	19,164	(89.5)	25,953	(86.9)
Percentage mental health benefit claimants in local community (mean, sd)	2	(1.6)	2.51	(1.7)	2.23	(1.6)	2.03	(1.5)
Percentage population of local community resident in NHS psychiatric establishment (mean, sd)	0	(0.3)	0.03	(0.4)	0.02	(0.3)	0.02	(0.3)
GP quality - % practice population with SMI with care plan (mean, sd)	1	(0.1)	0.84	(0.1)	0.85	(0.1)	0.84	(0.1)
GP access - % practice population able to see GP within 48 h (mean, sd)	1	(0.1)	0.82	(0.1)	0.82	(0.1)	0.83	(0.1)

### Data sources

Our study combined several datasets. Record-level data on hospital admissions were obtained from the Hospital Episodes Statistics (HES) which covers all NHS-funded secondary care in England. These data are reported as Finished Consultant Episodes (FCEs) and we converted these to continuous inpatient spells (CIPS) (admissions). Using CIPS has the advantage that it reduces coding errors e.g. where patients leave hospital for a weekend but are not discharged, they may otherwise be coded as a new admission on their return. We used HES to derive our dependent variable (LOS) and a range of demographic and clinical characteristics. Individual patient records were linked over time through a unique patient identifier, based on the patient’s NHS number. Data on local area-level characteristics (i.e. the number of people resident in an NHS community psychiatric establishment, and urban status) were sourced from the Office of National Statistics (ONS). These data were derived from the 2001 Census and were available at small area level (Lower Super Output Area (LSOA)). Data on the number of incapacity benefit claimants at small area level were obtained from the Department of Work and Pensions. Data on access to and quality of care for patients with SMI received in primary care were extracted from the Quality and Outcomes Framework (QOF) dataset and the GP Patient Survey (GPPS) dataset and linked to HES through the practice identifier and the year. Additional file 1: Appendix 2 provides a full list of datasets and sources. As confirmed by the University of York Research Ethics Committee, no ethical approval was required for this study since it is classed as low risk due to minimal burden or intrusion for participants as it is based on the analysis of anonymised secondary data.

### Data

LOS for each admission was calculated as the difference between the dates of admission to and discharge from hospital. All patients were admitted and discharged from the same hospital. Patients with unfinished episodes were dropped from the sample.

For each admission, we also extracted information from HES on socio-demographic variables such as age (we categorised patients’ age into seven 10-year bands and used the first band (18–24) as a reference category), gender, ethnicity, and carer support; clinical variables including main and secondary diagnoses, previous history of psychiatric care, legal status - whether the patient was detained under the Mental Health Act; and the mode of discharge (discharged by clinician, self-discharged, or died in hospital).

In relation to co-morbidity, previous studies adopt a range of different approaches, with many studies including co-morbidity in terms of secondary diagnoses of a mental health condition, rather than other clinical conditions. Some ignore this aspect completely [31]; others record whether a secondary diagnosis was present or absent [29]; and many tend to focus only on a secondary diagnosis related to substance or alcohol misuse or personality disorder [23, 35, 37].

We counted the total number of co-morbidities for a patient up to a maximum of 13, including secondary diagnoses for mental health and non-mental health conditions. We imposed a limit of 13 to account for the change in the number of available fields in HES for recording secondary diagnoses (ranging from 13 in 2006 to 19 in year 2010). We also derived a set of indicator variables for a secondary diagnosis of co-morbid alcohol and substance misuse (F10-F19) [35, 37] and co-morbid personality disorder (F60) [37].

We derived a number of neighbourhood level characteristics to account for the local context, e.g. the deprivation profile. We extracted data on the proportion of the local population who resided in NHS community psychiatric establishments. Ideally, we would have used a measure based on the number of beds available each year (rather than occupancy at one time point). However, as long as demand for community beds is at least equal to supply, the measure was considered a reasonable approximation of capacity and therefore a likely proxy for local area need. Socio-economic status was approximated by the percentage of the local population claiming incapacity benefit for a mental disorder. Since the LSOA population (i.e. denominator) changed over time, we estimated moving averages for both these variables. We then categorised the deprivation measure (i.e. incapacity claimants) into quintiles. Finally, we accounted for whether the local area was ‘urban’ (defined as having a population above 10,000), using a dummy variable based on the ‘Rural and Urban Area Classification for Super Output Areas, 2004’ (from ONS). This variable was assumed to be time-invariant.

Effective primary care may shorten patients’ LOS in two ways: firstly, if hospitals can be confident that the patient will be followed up by the GP practice they may decide to discharge the patient more quickly. Secondly, patients with better access to primary care prior to admission may require a shorter stay once admitted.

The Quality and Outcomes Framework (QOF) is a pay-for-performance scheme in primary care which includes a set of indicators for SMI against which practices score points according to their level of achievement. We extracted data on the proportion of SMI patients with a comprehensive care plan documented, which we interpreted as a measure of quality and continuity of care. To approximate accessibility of primary care services, we extracted the proportion of patients reported to have been seen by their GP within 48 h, derived from the annual GP survey. Both variables were measured at GP practice level and linked to the HES record through unique practice and year identifiers.

### Exclusions

We excluded admissions with very long LOS, defined as stays over 180 days (approximately 6 months), to reduce the effect of unusually long stay patients on the stability of the estimates and focus on a more homogeneous patient population that reflects the majority of cases seen in the inpatient setting. These long-stay patients tend to be different with respect to observable characteristics. For example, those patients staying longer than 180 days are twice as likely to be detained and 1.5 times as likely to have a main diagnosis of schizophrenia (ICD-10: F20). To ensure our analysis included all patients who could have stayed in hospital up to the upper threshold, we excluded admissions that occurred after the 2nd October 2010 calculated as 31^st^ March 2011 minus 180 days.

We also excluded admissions to mental health providers which treated fewer than 10 admissions for the particular clinical diagnosis sub-category over our study period (see study population). Finally, patients were excluded if they were recorded as living outside of England.

### Analysis

Poisson regression models were estimated to relate observed LOS to patient characteristics, neighbourhood characteristics and indicators of primary care. All models included hospital fixed effects to account for unobserved differences in hospital policies, efficiency, and case-mix. Hence, coefficients are estimated from within-hospital variation only. We included time fixed effects to account for common temporal trends. No exposure term was defined. Poisson regression was appropriate for these data due to the skewed distribution of LOS. It was also preferable to logarithmic transformations, which are commonly used to analyse LOS, because it estimated the conditional mean on the scale of interest and did not suffer from re-transformation bias [44, 45]. Poisson regression is increasingly used to analyse length of stay and cost data, and has been found to fit those data at least as well as for example, Weibull or Cox proportional hazard survival models [46, 47]. Since censoring was not a major concern in this study - only 2.7 % of patients self-discharged or died in hospital - we decided to model these factors as covariates. The Poisson estimator produces unbiased point estimates as long as the conditional mean is correctly specified. We obtained robust Huber-White standard errors to account for over-dispersion or other misspecification of the variance function [48].

Estimated effects are reported as average partial effects (APEs), which represent the expected change in LOS for a unit change in the independent variable. APEs were calculated conditional on hospital fixed effects, which we recovered after estimation using the procedure outlined in [48] (p.281). We also calculated Incidence Rate Ratios (IRRs) with two-sided 95 % confidence intervals, where values greater than 1 indicate an increase in relative risk of incurring an additional inpatient day.

All models were estimated on the pooled sample of all SMI admissions and separately for the three groups of SMI admissions. We compared the estimated effects across groups to explore heterogeneity in the effect of risk factors. We also correlated the hospital fixed effects estimates across groups to examine whether unobserved hospital characteristics had a similar effect on LOS for the different patient groups.

All analyses were conducted in Stata 13.

## Results

### Descriptive analysis

Our sample included 89,510 admissions for patients treated in 67 hospitals and who were registered with 7,792 GP practices. Across all five years, the median annual volume of admissions with a primary diagnosis of SMI was 270.

**Fig. 1:**
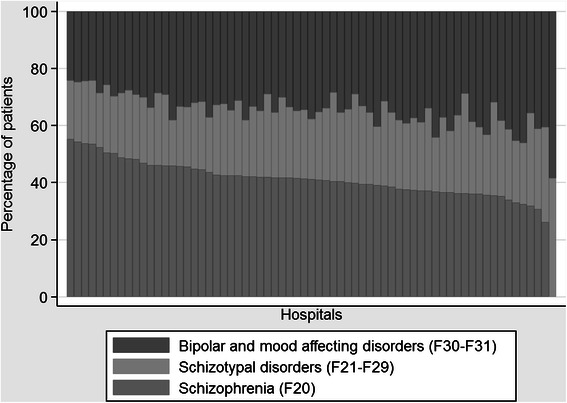
Variation in diagnosis by hospital provider, all years pooled

Approximately 42.7 % of admissions had a recorded primary diagnosis of schizophrenia, and another 33.4 % were diagnosed with bipolar disorder or a manic episode (Table 1). However, there was substantial variation in intake across providers. Figure 1 shows the proportion of patients in each of the three sub-groups by provider. For some providers, 55 % of the SMI patients were diagnosed with schizophrenia, whereas the proportion in other providers was less than 30 %. Similarly, the proportion of patients with bipolar or mood affective disorder was around 40 % (and one as high as nearly 60 %) in some providers, but was just over 20 % in other hospitals.

**Fig. 2:**
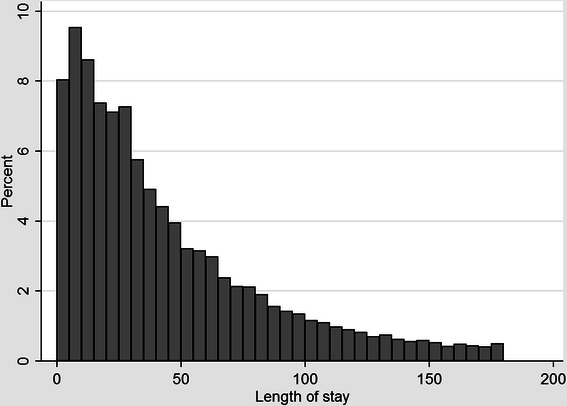
Histogram of length of stay, pooled across all diagnostic groups and years

**Fig. 3:**
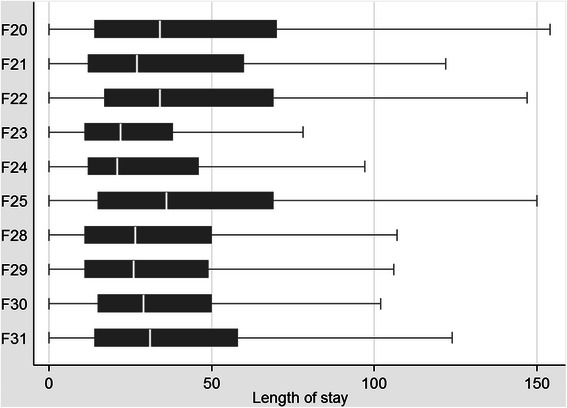
Boxplot of length of stay by diagnosis, all years pooled

Figure 2 shows a histogram of the distribution of LOS. LOS fell very slightly over time by on average around 0.2 to 0.4 days per year across the three sub-groups (Table 2) and LOS was longest for individuals with a main diagnosis of schizophrenia (F20) or schizoaffective disorder (F25) (Fig. 3).

**Table 2:** LOS by diagnostic group and pooled over time

	All (F20-F31) (1)		Schizophrenia (F20) (2)		Psychotic and schizoaffective disorder (F21-F29) (3)		Manic and bipolar disorder (F30-F31) (4)	
Financial year	Mean	SD	Mean	SD	Mean	SD	Mean	SD
2006/07	44.4	40.0	48.0	43.3	41.6	38.5	41.6	35.7
2007/08	43.3	39.7	47.0	42.7	40.8	38.5	40.2	35.9
2008/09	45.0	40.1	49.0	42.9	42.1	39.1	42.2	36.7
2009/10	43.7	39.6	47.7	42.7	40.6	37.8	41.1	36.3
2010/11	42.7	38.4	46.1	40.9	40.2	37.5	40.5	35.7
Pooled	43.9	39.7	47.7	42.7	41.1	38.3	41.2	36.1

### Estimation results - overview

Table 3 shows the average partial effects (APEs) estimates for the pooled model (column (1)) and then separately for the three types of SMI patient (columns (2) to (4)). Table 4 presents the results as Incidence Rate Ratios (IRR). In the pooled model, the majority of diagnostic groups had a shorter LOS than schizophrenia, some as much as 20 days shorter (F22). Diagnosis was a key predictor of LOS in the pooled model. Results were broadly consistent across the three diagnostic groups of patients. However, there were some differences in LOS across diagnoses: F23, F28 and F29 had significantly shorter LOS than schizotypal disorder (F21) of between 9 and 17 days. People with bipolar affective disorder had a significantly longer LOS of 7 days compared to those suffering from a manic episode (F30).

**Table 3:** Factors determining hospital length of stay – regression results, Average Partial Effects (APEs)

	Pooled (F20-F31) (1)		Schizophrenia (F20) (2)		Psychotic and schizoaffective disorder (F21-F29) (3)		Manic and bipolar disorder (F30-F31) (4)	
Variable	APE	SE	APE	SE	APE	SE	APE	SE
Main diagnosis								
Schizophrenia (F20)	(base category)		(base category)					
Schizotypal disorder (F21)	−4.16	0.71***			(base category)			
Persistent delusional disorder (F22)	−19.56	1.04***			−2.12	2.86		
Acute and transient psychotic disorder (F23)	−11.57	4.69*			−17.20	2.15***		
Induced delusional disorder (F24)	0.75	0.52			−9.34	5.65		
Schizoaffective disorders (F25)	−11.67	2.32***			3.78	3.18		
Other nonorganic psychotic disorders (F28)	−11.42	1.10***			−9.29	3.79*		
Unspecified nonorganic psychosis (F29)	−6.36	0.48***			−9.03	2.69***		
Manic episode (F30)	−3.02	2.80					(base category)	
Bipolar affective disorder (F31)	−12.57	1.01***					7.42	1.27***
Patient demographics and clinical characteristics								
Age 25-34	−1.63	0.61**	−1.72	0.81*	−0.93	1.13	−2.64	1.44
Age 35-44	−3.54	0.53***	−3.84	0.76***	−3.68	1.10***	−3.65	1.37**
Age 45-54	−2.25	0.59***	−3.22	0.98***	−2.25	1.00*	−0.66	1.42
Age 55-64	1.64	0.63**	−0.49	0.98	4.56	1.35***	4.31	1.80*
Age 65-74	10.88	1.23***	6.21	1.60***	14.39	2.33***	18.55	3.01***
Age 75 and over	18.64	1.57***	11.60	2.45***	25.57	3.84***	27.45	3.73***
Male	−0.41	0.38	−1.35	0.53*	−0.62	0.62	0.72	0.77
Detained	15.98	1.17***	19.48	1.81***	14.72	2.26***	16.51	1.76***
Ethnicity: mixed	2.31	0.99*	0.57	1.49	3.65	1.80*	7.74	3.45*
Ethnicity: Asian	0.69	0.64	0.68	0.82	1.92	1.42	−0.45	0.89
Ethnicity: black	4.46	0.63***	5.28	0.93***	3.99	1.25**	4.88	1.70**
Ethnicity: unknown or missing	−0.77	0.72	0.10	1.21	−0.81	1.17	−2.31	1.87
Patient has a carer	3.16	1.14**	3.19	1.35*	1.44	1.64	5.50	2.22*
Patient was previously treated for mental health issues	−1.00	0.76	−2.51	0.94**	0.15	0.94	0.41	1.22
MH benefit claimants - 2nd quintile	0.63	0.41	−0.07	0.62	1.12	0.94	1.32	0.75
MH benefit claimants - 3rd quintile	1.41	0.47**	0.59	0.67	1.24	1.00	3.14	0.97**
MH benefit claimants - 4th quintile	2.43	0.78**	1.41	0.99	1.75	1.28	5.76	1.09***
MH benefit claimants - 5th quintile	2.65	0.68***	1.11	0.88	3.03	1.34*	6.08	1.13***
Number of comorbidities	1.17	0.33***	1.04	0.35**	1.29	0.36***	1.53	0.53**
Alcohol and substance misuse	−4.21	0.67***	−4.96	1.05***	−2.40	1.38	−5.10	1.50***
Co-morbid personality disorder	−7.81	1.30***	−9.14	2.19***	−7.18	2.91*	−9.46	2.19***
Discharge								
Self-discharged	−19.99	1.85***	−19.24	2.48***	−20.37	3.11***	−29.17	2.76***
Died in hospital	−3.30	1.64*	−3.56	2.73	−0.96	4.12	−6.03	3.09
Access to care								
Urban	0.41	0.61	−0.10	0.91	0.67	1.02	1.20	1.06
% residents of local community in psychiatric establishment	−0.04	0.41	0.11	0.52	0.01	1.30	−0.41	0.87
Ability to access GP within 48 h	−0.54	1.12	0.10	1.73	−2.74	2.68	0.10	2.79
Care plan developed in primary care	−1.01	0.95	−2.18	1.57	2.92	2.16	−1.70	2.23
Time effects								
Year 2007	−1.18	0.97	−1.25	1.17	−1.27	1.45	−1.77	1.34
Year 2008	0.22	0.86	0.49	1.06	−0.44	1.19	0.43	1.37
Year 2009	−1.47	0.99	−1.34	1.33	−2.30	1.20	−1.79	1.33
Year 2010	−3.08	1.15**	−3.50	1.45*	−3.67	1.44*	−3.22	1.78
Pseudo-R^2^	0.061		0.046		0.091		0.050	
N	89,510		38,216		21,415		29,879	

Note: Evaluated at the mean of the estimated hospital effects. Interaction effects are subsumed into main effects. Pseudo-R^2^ are based on model with standard errors clustered at hospital level but no hospital fixed effects

* *p* < 0.05; ** *p* < 0.01; *** *p* < 0.001

**Table 4:** Factors determining hospital length of stay – regression results, Incidence Rate Ratios (IRRs)

	Pooled (F20-F31) (1)		Schizophrenia (F20) (2)		Psychotic and schizoaffective disorder (F21-F29) (3)		Manic and bipolar disorder (F30-F31) (4)	
Variable	IRR	95 % CI	IRR	95 % CI	IRR	95 % CI	IRR	95 % CI
Main diagnosis								
Schizophrenia (F20)	(base category)		(base category)					
Schizotypal disorder (F21)	0.91	(0.88; 0.94)			(base category)			
Persistent delusional disorder (F22)	0.64	(0.62; 0.66)			0.96	(0.84; 1.08)		
Acute and transient psychotic disorder (F23)	0.77	(0.62; 0.95)			0.69	(0.61; 0.78)		
Induced delusional disorder (F24)	1.02	(0.99; 1.04)			0.82	(0.63; 1.05)		
Schizoaffective disorders (F25)	0.77	(0.69; 0.85)			1.09	(0.96; 1.23)		
Other nonorganic psychotic disorders (F28)	0.77	(0.74; 0.81)			0.82	(0.68; 0.98)		
Unspecified nonorganic psychosis (F29)	0.87	(0.85; 0.88)			0.82	(0.72; 0.94)		
Manic episode (F30)	0.93	(0.82; 1.06)					(base category)	
Bipolar affective disorder (F31)	0.75	(0.72; 0.78)					1.14	(1.10; 1.18)
Patient demographics and clinical characteristics								
Age 25-34	0.99	(0.93; 1.04)	1.00	(0.91; 1.10)	1.01	(0.93; 1.10)	0.96	(0.89; 1.03)
Age 35-44	0.94	(0.90; 0.99)	0.95	(0.88; 1.03)	0.94	(0.86; 1.02)	0.95	(0.88; 1.02)
Age 45-54	0.99	(0.94; 1.03)	0.98	(0.91; 1.07)	0.98	(0.91; 1.05)	1.00	(0.93; 1.08)
Age 55-64	1.10	(1.05; 1.16)	1.06	(0.97; 1.15)	1.17	(1.07; 1.27)	1.12	(1.04; 1.21)
Age 65-74	1.32	(1.25; 1.39)	1.23	(1.12; 1.34)	1.40	(1.30; 1.52)	1.37	(1.26; 1.48)
Age 75 and over	1.50	(1.41; 1.60)	1.34	(1.22; 1.48)	1.63	(1.47; 1.81)	1.56	(1.41; 1.72)
Male	1.06	(1.00; 1.12)	1.04	(0.95; 1.13)	1.05	(0.96; 1.16)	1.06	(0.99; 1.14)
Detained	1.41	(1.35; 1.47)	1.52	(1.45; 1.60)	1.35	(1.28; 1.42)	1.31	(1.25; 1.37)
Ethnicity: mixed	1.07	(1.01; 1.13)	1.05	(0.97; 1.14)	1.09	(0.99; 1.19)	1.10	(0.99; 1.23)
Ethnicity: Asian	1.03	(0.99; 1.06)	1.04	(0.99; 1.09)	1.04	(0.97; 1.12)	1.01	(0.97; 1.05)
Ethnicity: black	1.12	(1.09; 1.15)	1.15	(1.10; 1.20)	1.11	(1.05; 1.17)	1.11	(1.04; 1.17)
Ethnicity: unknown or missing	0.99	(0.95; 1.03)	1.03	(0.96; 1.09)	0.97	(0.91; 1.04)	0.95	(0.88; 1.02)
Interaction: Detained + Ethnicity: mixed	0.94	(0.84; 1.06)	0.85	(0.74; 0.98)	0.98	(0.80; 1.20)	1.14	(0.92; 1.41)
Interaction: Detained + Ethnicity: Asian	0.95	(0.89; 1.02)	0.91	(0.83; 1.00)	1.00	(0.91; 1.11)	0.93	(0.83; 1.05)
Interaction: Detained + Ethnicity: black	0.93	(0.88; 0.98)	0.90	(0.85; 0.96)	0.91	(0.84; 0.99)	0.91	(0.84; 0.98)
Interaction: Detained + Ethnicity: unknown or missing	0.99	(0.92; 1.06)	0.91	(0.82; 1.01)	1.03	(0.92; 1.16)	1.05	(0.90; 1.22)
Patient has a carer	1.07	(1.02; 1.12)	1.07	(1.01; 1.13)	1.03	(0.96; 1.10)	1.10	(1.03; 1.17)
Patient was previously treated for mental health issues	0.98	(0.94; 1.01)	0.95	(0.91; 0.99)	1.00	(0.96; 1.04)	1.01	(0.97; 1.05)
MH benefit claimants - 2nd quintile	1.01	(1.00; 1.03)	1.00	(0.97; 1.03)	1.03	(0.99; 1.07)	1.02	(1.00; 1.05)
MH benefit claimants - 3rd quintile	1.03	(1.01; 1.06)	1.01	(0.98; 1.04)	1.03	(0.99; 1.07)	1.06	(1.02; 1.09)
MH benefit claimants - 4th quintile	1.06	(1.02; 1.09)	1.03	(0.99; 1.08)	1.04	(0.99; 1.09)	1.11	(1.07; 1.14)
MH benefit claimants - 5th quintile	1.06	(1.03; 1.09)	1.03	(0.99; 1.07)	1.07	(1.01; 1.13)	1.11	(1.07; 1.15)
Number of comorbidities	1.03	(1.01; 1.04)	1.02	(1.01; 1.04)	1.03	(1.01; 1.04)	1.03	(1.01; 1.05)
Alcohol and substance misuse	0.90	(0.88; 0.93)	0.89	(0.85; 0.93)	0.95	(0.89; 1.01)	0.91	(0.86; 0.96)
Co-morbid personality disorder	0.82	(0.77; 0.88)	0.80	(0.71; 0.90)	0.84	(0.73; 0.97)	0.84	(0.77; 0.91)
Discharge								
Self-discharged	0.55	(0.49; 0.62)	0.57	(0.50; 0.66)	0.56	(0.48; 0.66)	0.50	(0.44; 0.57)
Died in hospital	0.93	(0.86; 1.00)	0.92	(0.81; 1.05)	0.98	(0.82; 1.17)	0.90	(0.80; 1.01)
Access to care								
Urban	1.01	(0.98; 1.04)	1.00	(0.96; 1.04)	1.01	(0.97; 1.06)	1.02	(0.99; 1.06)
% residents of local community in psychiatric establishment	1.00	(0.98; 1.02)	1.00	(0.98; 1.03)	1.00	(0.95; 1.06)	0.99	(0.96; 1.02)
Ability to access GP within 48 h	0.99	(0.94; 1.04)	1.00	(0.93; 1.08)	0.94	(0.83; 1.06)	1.00	(0.91; 1.10)
Care plan developed in primary care	0.98	(0.94; 1.02)	0.95	(0.89; 1.02)	1.07	(0.98; 1.16)	0.97	(0.90; 1.05)
Time effects								
Year 2007	0.97	(0.93; 1.02)	0.97	(0.92; 1.02)	0.97	(0.92; 1.03)	0.97	(0.93; 1.02)
Year 2008	1.00	(0.97; 1.04)	1.01	(0.97; 1.06)	0.99	(0.94; 1.04)	1.01	(0.96; 1.05)
Year 2009	0.97	(0.92; 1.01)	0.97	(0.92; 1.03)	0.95	(0.91; 1.00)	0.97	(0.93; 1.01)
Year 2010	0.93	(0.88; 0.98)	0.92	(0.86; 0.99)	0.92	(0.87; 0.98)	0.95	(0.89; 1.01)
Pseudo-R^2^	0.061		0.046		0.091		0.050	
N	89,510		38,216		21,415		29,879	

Note: Model includes hospital fixed effects (not shown). Age x gender interactions suppressed. Pseudo-R^2^ are based on model with standard errors clustered at hospital level but no hospital fixed effects

### Estimation results – individual characteristics

Our findings suggest that most independent risk factors do not have a differential effect for different diagnostic sub-groups. However we do note some heterogeneity in the effects. In terms of patient demographics and clinical characteristics, we found an age gradient with patients from age 65 and above with schizophrenia, and from age 55 and above for the other diagnostic subgroups and in the pooled model, exhibiting progressively longer LOS compared to 18–24 year-olds. This age gradient for the 65 to 74-year old age group, relative to the 18 to 24-year old age group, was 11 days in the pooled model and ranged from 6 days for the schizophrenia subgroup, 14 days for schizoaffective disorder and 19 days for bipolar disorder. Gender was not a significant predictor of LOS. Longer LOS was associated with formal detention (16 days in the pooled model and between 15 days for schizoaffective disorder and 19 days for schizophrenia) and with black ethnicity (around 4 days), although detained patients with black ethnicity had shorter LOS than detained white patients (see interactions in Table 4). Having an informal carer was associated with longer LOS in the pooled model (3 days) although this was not significant in all models (2) to (4). Patients with schizophrenia who had a previous psychiatric history had a shorter LOS of around 2.5 days, but this was not the case in the pooled model or for any of the other sub-groups. In the pooled model, patients from more deprived neighbourhoods had a longer LOS (between 2 and 3 days) and the effect was larger in patients with bipolar disorder (6 days). Having a higher number of physical and psychiatric comorbidities was associated with longer LOS (1 day) while shorter LOS was associated with co-morbid substance or alcohol misuse (between 4 and 5 days), and co-morbid personality disorder (between 7 and 9 days) for all types of patient. Patients who decided to self-discharge had shorter LOS (between 19 and 29 days). Patients whose usual place of residence was an urban area did not have significantly different LOS compared with other patients. No association was found between LOS and primary care in terms of either access or quality variables.

### Hospital variation

**Fig. 4:**
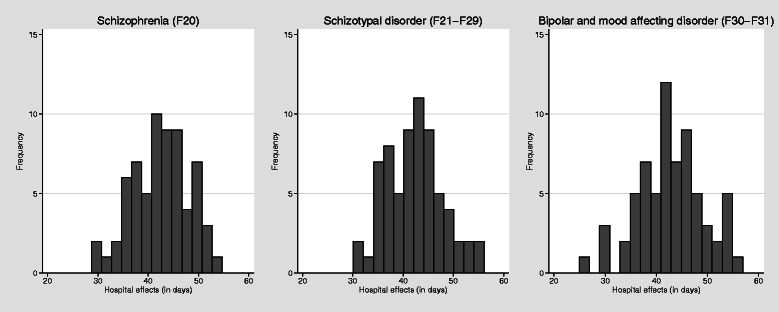
Boxplot of hospital effects, by diagnostic group

Figure 4 shows histograms of the estimated hospital fixed effects by diagnostic group. These fixed effects could be interpreted as the predicted length of stay for a given patient (here given by the reference category in Table 3). The median hospital effects were 42.8 days (Interquartile range (IQR) = 38.5 - 45.7) for schizophrenia (F20), 42.6 days (IQR = 38.0 - 46.0) for schizotypal disorders (F21-F29), and 42.3 days (IQR = 38.9 - 46.5) for bipolar and mood affective disorders (F30-F31). The differences amongst hospital fixed effects reflect the average effect on hospital LOS of differences across hospitals in factors that we do not observe.

The correlations between the hospital effects for the three sub-groups of patients were high (rho > 0.75) for all pairs of diagnostic groups.

## Discussion

To our knowledge, this is the first study to use large-scale national routine data to examine the key determinants of LOS for particular patient sub-groups with serious mental illness in England. Previous literature has tended to produce inconsistent results about factors associated with LOS partly because of small sample sizes and also due to the limitations of the methods employed in some studies. Our main contribution to the existing literature is in terms of our methodology which, compared to other studies, provides results which are more robust. The methodological advances include estimating a Poisson regression model with hospital fixed effects, rather than using a logit model to examine long-stay patients using an arbitrary cut-off point to model case-controls, and taking account of LOS as a continuous variable. Where many previous studies ignore hospital effects, we examined differences in LOS between mental health providers. Our larger sample size enabled us to improve on previous studies by estimating separate models for three key diagnostic sub-groups to analyse the independent contribution of a range of potential determinants of LOS on each of the broad classes of diagnoses. Our study population was everyone admitted to an NHS mental health hospital in England with SMI over the period 2006 to 2010 and was considerably larger and more representative than previous studies. There are no reliable estimates of the number of patients seeking care in the private sector, but this is likely to be small as the vast majority of mental health hospital care in England is publicly funded. Specifically, the £143 m market for privately funded mental health hospital care [49] compares with £2 billion of NHS spending on psychotic disorders [50].

Contrary to some previous studies, we found that diagnosis was a strong predictor of LOS [40, 51]. We found that shorter LOS was associated with co-morbid substance or alcohol misuse, and with co-morbid personality disorder, although recorded prevalence of these co-morbidities may be low due to poor coding. This finding is however consistent with previous literature and may be because when these patients’ symptoms resolve following inpatient detoxification, they are more likely to leave against medical advice (self-discharge), and may be motivated to show improvement so they can leave to regain access to drugs or alcohol [35, 37]. Indeed patients who self-discharged had shorter LOS. It may also reflect the transient nature of psychotic symptoms in the context of substance misuse, where there is more rapid resolution upon admission to hospital and removal from the usual environment. While previous literature has been inconsistent with respect to the association with age, reporting positive [30, 33, 35], and negative findings [29, 31, 36]), in our study we found a strong age gradient only for people aged 55 and above (and the effect was not apparent until 65 for those with schizophrenia). We also found, as in previous literature [37, 38], that compulsory admission was positively associated with LOS, increasing it by 16 days overall (19 days for schizophrenia, 15 days for schizoaffective disorder and 17 days for bipolar disorder). While studies have found mixed results on the association between male gender and LOS (positive [24, 30, 31], negative [37]), gender was not a significant predictor of LOS in our analyses. Previous evidence on the association between co-morbidities and LOS has been inconsistent: while some studies found that patients with more co-morbidities had longer LOS [24, 29], others found that individuals with no comorbidity had longer LOS [30]. Our study found that having a higher number of psychiatric and physical comorbidities was associated with longer LOS of around 1 day. Some previous studies have reported positive associations between prior hospitalisation and LOS [35, 38] and others found a negative relationship [29]; in our analyses, only schizophrenia patients with a psychiatric history had a shorter LOS of around 2.5 days. This may be because these patients are well known to services and crisis stabilisation can be achieved more swiftly since relapse signatures will be familiar, medication regimes will be tried and tested, and care plans are more likely to be in place.

Having a carer was associated with longer LOS overall in the pooled model and for schizophrenia and bipolar disorder patients, but there was no effect for schizoaffective disorder patients. It is possible that if carers experience a significant carer burden from patients with high levels of need, LOS may be prolonged, in the interests of protecting carers’ health and wellbeing. Just less than 7 % of patients have an unpaid carer registered in their hospital record. The record may underestimate the actual level of both formal and informal care that this patient population receive. If a record of having a carer is associated with increased patient need, then this may explain the positive association that we observe.

Patients with manic or bipolar disorders who were from more deprived neighbourhoods had longer LOS whilst this was not the case for schizophrenia patients.

Although there were similarities in the association between LOS and patient characteristics across the three diagnostic patient groups, there were some noticeable differences. Whilst these should be interpreted with caution, our results suggest that there may be advantages to modelling LOS stratified by diagnostic groupings to more accurately determine associations between case-mix which can be used to ensure prospective payment systems reflect accurately the resource use within sub-groups.

We found a large degree of variation in case-mix between providers. This will likely have implications for the costs imposed on them by the risk profile of their patient population, particularly if hospitals predominantly treat older patients with complex care needs and detained patients. We also found significant variation in the hospital fixed effects within diagnostic groupings. The interquartile range of the hospital fixed effects for each diagnostic group is around 9 days suggesting a significant spread in the distribution and large differences between providers in the unexplained variation in LOS. We also found a high correlation between the provider effects across the different diagnostic groups. This suggests that hospitals with unexplained high LOS for one diagnostic group will also have high LOS for another sub-group. These hospitals may be systematically different in the way they manage and treat patients. Unobserved hospital characteristics (such as the quality of care, quality of management, unmeasured differences in average case-mix, or differences in efficiency) therefore appear to have similar effects on LOS for different types of patients.

The proposed NTPS for mental health providers is based on need and, other than assigning patients to the super-classes of non-psychotic, psychosis and organic mental illness, the system does not directly use diagnoses (ICD-10 codes) to cluster service users. The Mental Health Clustering Tool, used to allocate service users to the 21 clusters, explicitly states that people with the same diagnosis can be assigned to different clusters, and that individuals can move between clusters as their needs change over time [52]. Our results suggest that the payment system may need to be tailored according to diagnostic group. A prospective payment system should be fair (e.g. paying the same for treating patients with similar needs), but also needs to take account of factors beyond the control of a hospital (e.g. the characteristics of patients such as diagnosis if this affects LOS, age, detention status, local input prices). However, a balance needs to be struck. If some factors make little economic difference, though statistically significant, they should not be used in the payment system as they would add unnecessary complexity. There are also risks of unintended consequences if some diagnoses or detention status attract a higher payment, generating inappropriate incentives. Finally, the argument for paying by diagnosis hinges on the assumption that these are well coded. There are therefore concerns about the feasibility of implementing such a system (coding quality, gaming, etc.).

## Conclusions

This study used national administrative data linked to publicly available datasets to produce a large sample with a rich set of potential determinants of LOS for patients with SMI. Our data on individual patients was more limited than in studies adopting retrospective case note review but were comprehensive in that they covered all publicly funded hospital admissions in England. Many of the commonly identified risk factors were captured, although some were an imperfect match for those identified in the literature review. Other factors were omitted entirely due to limited data availability, including psychiatric functioning or severity, the use of seclusion or restraint and psychopharmacological medication. We also did not account for readmissions which may be important in relation to LOS and payment mechanisms, since providers with shorter LOS may benefit from early discharge, and a subsequent new admission for which they could be paid, unless incentives were put in place to discourage a quicker and sicker ‘revolving door’ phenomenon [53].

We found substantial variation between providers in unobserved hospital characteristics (such as differences in management culture or efficiency). Providers appear to be systematically different in terms of their resource use and this will likely result in some hospitals being ‘winners’ and others ‘losers’ under a prospective payment system. International experience suggests large variations in provider effects with respect to costs or LOS may make a classification system unsuitable for provider payment [13] as it may destabilize local health economies. There is therefore a need for a careful transition to any new payment system.

The variation in case-mix which we observed may be the result of genuine differences in risk profiles between providers, but may also be due to inconsistent use of diagnostic codes between providers. There are some limitations to the use of diagnostic classifications in HES for psychiatric admissions. Diagnostic coding may be done by staff removed from the nuances of psychiatric diagnosis, rather than by the rigorous application of ICD-10 criteria by clinicians. Whilst we have argued that payment systems may need to be tailored to diagnostic groupings, this would require the consistent and accurate use of diagnostic codes across mental health providers. Whilst some mental health professionals are reluctant to label patients, in part due to stigma, and argue for treating the person rather than the illness [54], diagnostic coding can be helpful to patients, by providing appropriate treatments and access to support and services including benefits [55]. A quality indicator has been recommended for use by commissioners and providers in drawing up contracts as part of the NTPS which incentivises the collection of a valid ICD-10 code [56]. Improved data quality on diagnostic coding is imperative for future research purposes to better understand the role of diagnosis as a driver of LOS and resource use as part of a funding system.

Challenges in future may be not just to reward hospitals properly but also to incorporate incentives for appropriate primary, community and social care to form part of the care package for individuals with SMI, moving towards personalised funding. Future research should therefore focus on examining cost drivers across the full range of services that SMI patients utilise and across the full patient care pathway. This will support the design and reimbursement of more effective and efficient care pathways. Inpatient LOS for SMI patients will remain an expensive but important component of that pathway and therefore understanding the key determinants of LOS is vital as mental health service commissioners and providers grapple with the challenges of continued cost pressures.

## Additional file

Additional file 1: Appendix 1. Literature review search strategy. **Appendix 2**. Data sources. (DOCX 33 kb)

## Abbreviations

APE: Average Partial Effect
BPRS: Brief Psychiatric Rating Scale
CIPS: continuous inpatient spell
GAF: Global Assessment of Functioning
GPPS: GP Patient Survey
HES: Hospital Episode Statistics
ICD-10: International Classification of Diseases 10th revision
IQR: Interquartile range
IRR: Incidence rate ratio
LOS: Length of stay
LSOA: Lower super output area
NHS: National Health Service
NTPS: National Tariff Payment System
ONS: Office for National Statistics
PbR: Payment by Results
QOF: Quality and Outcomes Framework
SMI: Serious mental illness
