# Supplementary material for: Determinants of hospital length of stay for people with serious mental illness in England and implications for payment systems: a regression analysis
Source: BMC Health Serv Res. 2015 Sep 30;15:439. doi: 10.1186/s12913-015-1107-6 (PMC4590310; doi:10.1186/s12913-015-1107-6)
Supplement: Additional file 1: Appendix 1. — Literature review search strategy. Appendix 2. Data sources. (DOCX 33 kb) [file 12913_2015_1107_MOESM1_ESM.docx]

# Additional file

### Appendix 1 – Literature review search strategy

Database: Ovid MEDLINE(R) In-Process & Other Non-Indexed Citations and Ovid MEDLINE(R) <1946 to Present>

Search Strategy:

1 exp schizophrenia/ (86224)

2 exp psychotic disorders/ (38857)

3 exp bipolar disorder/ (32004)

4 1 or 2 or 3 (139784)

5 *"length of stay"/ (7488)

6 *hospitalization/ (25667)

7 (hospitalis$ or hospitaliz$).ti. (28344)

8 5 or 6 or 7 (50295)

9 4 and 8 (2173 records)

The 2 concepts of length of stay and serious mental illness are combined here

10 exp Cohort Studies/ (1386410)

11 cohort$.tw. (292014)

12 controlled clinical trial.pt. (89633)

13 epidemiologic methods/ (30158)

14 10 or 11 or 12 or 13 (1607472)

15 9 and 14 (636 records)

Records for cohort studies and length of stay/mental illness

16 systematic$ review$.ti,ab. (59902)

17 meta-analysis as topic/ (13972)

18 meta-analytic$.ti,ab. (3789)

19 meta-analysis.ti,ab,pt. (73869)

20 metanalysis.ti,ab. (131)

21 metaanalysis.ti,ab. (1095)

22 meta analysis.ti,ab. (56652)

23 meta-synthesis.ti,ab. (243)

24 metasynthesis.ti,ab. (130)

25 meta synthesis.ti,ab. (243)

26 meta-regression.ti,ab. (2518)

27 metaregression.ti,ab. (290)

28 meta regression.ti,ab. (2518)

29 (synthes$ adj3 literature).ti,ab. (1440)

30 (synthes$ adj3 evidence).ti,ab. (4212)

31 integrative review.ti,ab. (931)

32 data synthesis.ti,ab. (7526)

33 (research synthesis or narrative synthesis).ti,ab. (773)

34 (systematic study or systematic studies).ti,ab. (7705)

35 (systematic comparison$ or systematic overview$).ti,ab. (1900)

36 evidence based review.ti,ab. (1312)

37 comprehensive review.ti,ab. (6983)

38 critical review.ti,ab. (11177)

39 quantitative review.ti,ab. (479)

40 structured review.ti,ab. (485)

41 realist review.ti,ab. (58)

42 realist synthesis.ti,ab. (38)

43 or/16-42 (158791)

44 review.pt. (1917285)

45 medline.ab. (60436)

46 pubmed.ab. (33675)

47 cochrane.ab. (32875)

48 embase.ab. (31680)

49 cinahl.ab. (10797)

50 psyc?lit.ab. (914)

51 psyc?info.ab. (13188)

52 (literature adj3 search$).ab. (26969)

53 (database$ adj3 search$).ab. (25071)

54 (bibliographic adj3 search$).ab. (1297)

55 (electronic adj3 search$).ab. (9078)

56 (electronic adj3 database$).ab. (11040)

57 (computeri?ed adj3 search$).ab. (2662)

58 (internet adj3 search$).ab. (1751)

59 included studies.ab. (7302)

60 (inclusion adj3 studies).ab. (6739)

61 inclusion criteria.ab. (37455)

62 selection criteria.ab. (20932)

63 predefined criteria.ab. (1122)

64 predetermined criteria.ab. (760)

65 (assess$ adj3 (quality or validity)).ab. (42430)

66 (select$ adj3 (study or studies)).ab. (39170)

67 (data adj3 extract$).ab. (30558)

68 extracted data.ab. (7231)

69 (data adj2 abstracted).ab. (3343)

70 (data adj3 abstraction).ab. (868)

71 published intervention$.ab. (109)

72 ((study or studies) adj2 evaluat$).ab. (108402)

73 (intervention$ adj2 evaluat$).ab. (6293)

74 confidence interval$.ab. (230417)

75 heterogeneity.ab. (96113)

76 pooled.ab. (46510)

77 pooling.ab. (7895)

78 odds ratio$.ab. (153324)

79 (Jadad or coding).ab. (123447)

80 or/45-79 (833186)

81 43 or 80 (907038)

82 9 and 81 (134 records)

Records for systematic reviews and length of stay/mental illness

83 15 or 82 (704 records)

Records for cohort studies or systematic reviews and length of stay/mental illness

### Appendix 2 – Data sources

| **Dataset / Website** | **Description** | **Availability** | **Variables based on the dataset** |
| --- | --- | --- | --- |
| Hospital Episode Statistics [HES] *  <http://www.hscic.gov.uk/hes> | Annual data on hospital inpatient admissions  Individual level | Held by HSCIC and released under a DSA** | Psychiatric admissions  Individual patient clinical and socio-demographic characteristics  Sensitive data on detentions, legal status, informal carer ** |
| Annual Quality and Outcomes Framework (QOF) data <http://qof.hscic.gov.uk/> | Annual counts of patients meeting /not meeting/ exception reported from QOF indicators  Practice level | Freely available | Achievement and exception QOF indicators for SMI |
| General Practice Patient Survey (GPPS)  <https://gp-patient.co.uk/surveys-and-reports> | Annual survey of sample of registered patients in each GP practice  Practice level | Freely available | 48 hour access to GP |
| Office for National Statistics (ONS)  <http://www.neighbourhood.statistics.gov.uk> | Large dataset including variables from the Census (2001) Small area (neighbourhood) level (LSOA) | Freely available | NHS community psychiatric residents |
| Incapacity Benefit data (Department for Work and Pensions)  <http://tabulation-tool.dwp.gov.uk/NESS/BEN/ibsda.htm> | Quarterly counts of claimants, by benefit type, age, gender, duration of claim and main disabling condition.  Small area (neighbourhood) level (LSOA) | Freely available | Deprivation |
| **Notes**: DH: Department of Health (England); DSA: Data Sharing Agreement; HSCIC: Health & Social Care Information Centre; LSOA: lower super output area  * HES Copyright © 2006-2010, re-used with the permission of The Health and Social Care Information Centre. All rights reserved  ** Under a DSA, the dataset is released on condition that it is not shared with any third party. | | | |
